# Trajectories of Identity Formation Modes and Their Personality Context in Adolescence

**DOI:** 10.1007/s10964-018-0824-7

**Published:** 2018-03-01

**Authors:** Ewa Topolewska-Siedzik, Jan Cieciuch

**Affiliations:** Cardinal Stefan Wyszyński University in Warsaw, Warsaw, Poland

**Keywords:** Identity formation modes, Identity development, Personality, Early and middle adolescence, Longitudinal research, Latent growth curve modeling

## Abstract

Identity formation is a dynamic process during adolescence. Trajectories of identity formation were assessed longitudinally in early and middle adolescents, taking into account the personality underpinnings of this process. Identity formation was conceptualized according to the circumplex of identity formation modes. The model distinguishes basic modes rooted in Marcia’s categories of exploration and commitment. Plasticity and stability, the two higher order Big Five meta-traits, were used to assess personality underpinnings. This study includes five measurement waves over 1.5 years and involves 1,839 Polish participants; 914 early adolescents (53.9% girls) and 925 middle adolescents (63.8% girls). The results suggest that (1) the four identity formation modes change dynamically, showing linear and curvilinear growth and that (2) identity formation mode trajectories are more dynamic in middle adolescence than in early adolescence. The results also showed that, in the conditional model, (3) the higher-order personality factors and gender affect the growth factors of identity formation modes. Overall, trajectories of identity formation modes are more linear during early adolescence and more curvilinear during middle adolescence. The initial levels in identity trajectories are influenced by the personality metatraits but only plasticity is related to change among early adolescents.

## Introduction

Identity formation is considered to be one of the most important developmental tasks in adolescence. Both Erikson (1959, 1968) and contemporary scholars regard the process of identity development during adolescence as dynamic; yet longitudinal research still is needed to examine various trajectories of this process (Arnett 2015; Crocetti 2018; Klimstra et al. 2010; Kroger 2017; Kroger et al. 2010; Meeus 2011). Although the traditional framework for identity formation research was originally developed by Erikson (1959) and Marcia (1966), there are currently several more recent models that can be considered extensions of Marcia’s paradigm (e.g., Berzonsky 1989; Crocetti et al. 2008; Luyckx et al. 2008; McLean and Syed 2015; Schwartz et al. 2011). While the number of recent models has led to an accumulation of detailed knowledge on specific elements of the identity formation process, it has also resulted in a fragmentation of this knowledge due to a number of model-specific findings. Recently, the *circumplex of identity formation modes* (CIFM, Cieciuch and Topolewska 2017) has been developed as an attempt to integrate the various identity formation constructs and models by using Marcia’s redefined categories and linking them to recent developments in personality psychology in a systematic way. The aim of the current article is to describe the trajectories of identity formation modes and their personality underpinnings among early and middle adolescents in a theory-driven way consistent with the assumptions of the circumplex model of identity formation modes.

### Identity Development within Marcia’s Framework

To describe how people deal with identity-related issues and how they seek answers to the question “Who am I?”, Marcia (1966) proposed two categories of identity formation: *exploration* and *commitment*. Exploration refers to searching and experimenting with possibilities, while commitment is defined as making identity-relevant decisions. Further, Marcia (1966) distinguished four statuses of identity based on the two categories of exploration and commitment: achievement (commitment after exploration), moratorium (exploration without commitment), foreclosure (commitment without exploration), and diffusion (absence of both exploration and commitment). Scholars have since extended Marcia’s model proposing distinctions between various facets of exploration and commitment or statuses of which three models are especially predominant (i.e., Crocetti et al. 2008; Luyckx et al. 2006b, 2008). First, the three-dimensional model of identity formation processes by Crocetti et al. (2008) distinguishes *in-depth exploration*, *commitment* and *reconsideration of commitment* in various identity formation domains. Second, the model of five identity formation dimensions by Luyckx et al. (2008) consists of three kinds of exploration, *exploration in breadth, exploration in depth and ruminative exploration*, and two aspects of commitment, *commitment making and identification with commitment*. Lastly, the identity formation model by Berzonsky (1989) distinguishes three styles, informational, normative and diffuse-avoidant, as social-cognitive strategies of identity formation for the four statuses.

Unquestionably, the variety of different identity formation models yields detailed knowledge about identity formation. However, it also results in the fragmentation of the collected information as well as in a disconnect between particular theoretical approaches to identity research. However, at the same time, there are also attempts in the literature, both theoretical and empirical, to integrate constructs from different models or different aspects of identity formation (Cieciuch and Topolewska 2017; Crocetti et al. 2013; Galliher et al. 2017; Lile 2013). One of these proposals aims at a theoretical synthesis of the three above mentioned extensions of Marcia by means of the *circumplex of identity formation modes* (Cieciuch and Topolewska 2017). We use this model as the theoretical basis for our research.

### The Circumplex of Identity Formation Modes

The aim of the circumplex of identity formation modes (Cieciuch and Topolewska 2017; Topolewska and Cieciuch 2017) is to integrate various identity formation constructs developed within the Erikson-Marcia tradition. The three-dimensional model (Crocetti et al. 2008), the model of five identity formation processes (Luyckx et al. 2008) and Berzonsky’s (1989) model of identity formation styles were analyzed to find common ground where a synthesis of concepts of identity construction is possible. Cieciuch and Topolewska (2017) indicated the following aspects as being of particular importance in the integration of identity formation models: (1) the alignment of the categories currently used in research on identity formation, (2) a redefinition of the Marcia’s (1966) classical categories of exploration and commitment with regard to current social and cultural conditions, and (3) the location of identity formation constructs within the broader structure of personality. We briefly present the basic theoretical assumptions of the model below.

#### Identity formation mode

The list of currently used categories of identity formation constructs includes the following: processes or dimensions (Crocetti et al. 2008; Luyckx et al. 2008; Marcia 1966; Whitbourne et al. 2002), statuses (Marcia 1966) and styles (Berzonsky 1989; Marcia 1980). To reconcile various conceptual categories, especially viewed from the perspective of dimensional individual differences (i.e., styles, processes or dimensions) and types (i.e., statuses), Cieciuch and Topolewska (2017) justified the proposal of using the term of *mode*. In the identity formation circumplex, the basic descriptive category is an *identity formation mode*, defined as a manner of identity management typically implemented while dealing with identity-relevant issues.

#### Exploration and commitment in the current social reality

Undoubtedly, since the 1960s and Marcia’s (1966) first works, social reality has changed significantly. This change has happened in many ways including the number of possibilities with regard to exploration and experimentation, the period when identity is typically constructed (Kroger 2017) and the socially accepted stability of commitments made (Brown et al. 2002; Pinquart and Silbereisen 2005). Additionally, in current society, adolescents are exposed to new phenomena such as globalization (Barbieri et al. 2014), terrorism (Meeus 2015; Schwartz et al. 2009) or technological development (Carter and Grover 2015) that influence the current forms of exploration and commitment. In light of the above-mentioned issues, using the concepts of exploration and commitment as defined in the original work by Marcia or theory by Erikson may be problematic. As a result, refinement of these concepts was proposed by the circumplex of identity modes (Cieciuch and Topolewska 2017; Topolewska and Cieciuch 2017).

#### Identity modes within personality

Developed in the Erikson-Marcia tradition, the circumplex of identity formation modes takes knowledge of personality structure into account and places identity formation modes within the broader context of human personality. As postulated in the literature (Hatano et al. 2016; Wilt et al. 2011), commitment and exploration can be viewed as being conditioned by higher-order factors of personality, namely, *alpha* vs. *beta* or *stability* vs. *plasticity* (DeYoung et al. 2002; Digman 1997), which are sometimes named the two-factor model of personality (Cieciuch and Strus 2017). Stability consists of conscientiousness, agreeableness, and emotional stability, while plasticity is a constellation of extraversion and openness to experience. Stability, which maintains the cohesion and continuity of personality, can be viewed as a dispositional underpinning of commitment, which, in turn, is responsible for the stability of identity structure. Plasticity is a subsystem striving for activity and personal growth, similar to exploration in identity structure (see also Hatano et al. 2016; Wilt et al. 2011). From a theoretical perspective, personality and identity formation constructs can be located at different levels of the three-layer model of personality proposed by McAdams (Lilgendahl 2015; McAdams 1995; McAdams and Manczak 2011; McAdams and Pals 2006). At the very core is the layer of *dispositional traits*, consisting of somewhat stable constructs such as personality traits or higher-order personality factors, which can provide foundations for other constructs i.e., self-concept, attitudes, and goals. These other features, largely determined by the foundational traits, are located within the second layer containing *characteristic adaptations*, defined as constructs that are the result or manifestation of interactions between basic dispositional traits and environmental conditions. Identity formation dimensions, styles and modes can be located therein as constructs concerned with the identity management by means of the cognitive and social processing of values, attitudes, goals, and beliefs. The third layer of the personality system is *narrative identity*, which concerns the lifelong task of creating a comprehensive and meaningful story of life. In this way, identity formation constructs are located in the human personality structure and are underpinned by two basic personality metatraits.

#### Identity modes differentiated in the circumplex of identity formation modes

In line with Marcia (1966) and Berzonsky (1989), identity is defined in the circumplex of identity formation modes as a self-constructed, complex cognitive structure consisting of the subjectively chosen components that the individual deems relevant to who he or she is. The model proposes two basic dimensions: socialization vs. defiance and exploration vs. petrification (for the definitions, see Table 1). These dimensions, on the one hand, resemble the two higher-order factors of personality and, on the other hand, are similar to commitment and exploration, respectively, but also take into account the contemporary conditions of identity formation that are different from those in the era of Erikson and Marcia. Based on these two bipolar dimensions, the circumplex of identity formation modes distinguishes eight identity formation modes. The two other dimensions are *consolidation* (combination of socialization and exploration) vs. *diffusion* (combination of defiance and petrification) and *moratorivity* (combination of exploration and defiance) vs. *normativity* (combination of socialization and petrification), which can be treated as a combination of the two basic dimensions and thus are not taken into account in the current study.

**Table 1:** Four basic identity modes distinguished in the CIFM

Mode	Description
Socialization	Defining oneself in such a way as to perform one’s life roles well, according to the current stage in one’s life. Beliefs concerning oneself form a coherent and stable system associated with a sense of being in the right place
Exploration	An active involvement, agency in building an identity structure and solving identity-relevant dilemmas and problems. The focus is on probing one’s possibilities and testing whether a given activity is suitable for oneself
Defiance	The belief that one has not found one’s place in life. Because this mode is located between diffusion (identity indetermination) and moratorivity (desire to undertake a commitment), it poses the risk that the adopted commitment will be in stark opposition to social norms
Petrification	A lack of interest in thinking about oneself and developing an identity structure. The characteristic feature is fragmentation of a rather poorly developed cognitive identity structure, with the fragmented elements being rigid or even frozen

*Source*: Topolewska and Cieciuch (2017)

Either pole of the basic dimensions is conceptualized as a separate, qualitatively different variable. This conceptualization means that petrification and defiance are not simple oppositions to exploration and socialization, respectively. This kind of construct definition makes it possible to assess the basic identity formation categories in a more detailed way, which is particularly important in longitudinal research evaluating changes in the course of development. For example, an increase in the level of exploration does not have to mean a decrease in the level of petrification. One variable may rise with the other remaining at the same level.

The integrative power of the CIFM has already been empirically confirmed by Topolewska and Cieciuch (2017). This study encompassed constructs from the three-dimensional model of Crocetti et al. (2008), the five identity dimensions of Luyckx et al. (2008) and the three processing styles of Berzonsky (1989). The results showed that in-depth exploration (from the Crocetti et al. 2008 model), exploration in breadth (from the Luyckx et al. 2008 model) and informational style (Berzonsky 1989) are most closely connected to the exploration identity formation mode and that commitment making and identification with commitment from Luyckx et al. (2008) and commitment from Crocetti et al. (2008) have the strongest relationship with the socialization identity formation modes. While the petrification mode is not a unique equivalent of any variable, the defiance mode is strongly associated with reconsideration of commitment from Crocetti et al. (2008).

### Empirical Findings on Personality Underpinnings of Identity Development

In the literature, the relationship between identity and personality constructs has been studied in both theoretical and empirical ways, with data-driven research being the prevalent approach. Empirical studies have addressed the personality profiles of identity statuses (Crocetti et al. 2008), the interplay between identity and personality variables (Hatano et al. 2016; Luyckx et al. 2014), and the relationships between these two groups of constructs at three levels of personality generality: higher-order factors, personality traits, and personality facets (Dunkel et al. 2008; Duriez et al. 2004; Klimstra et al. 2013; Klimstra et al. 2013; Wilt et al. 2011).

The results from the literature show a pattern of the relationships between identity formation constructs and personality traits. Studies concerning the identity processes proposed in the latest identity formation models (Hatano et al. 2016; Klimstra et al. 2012; Klimstra et al. 2013; Luyckx et al. 2012, 2014) show that exploration in depth is often associated with low emotional stability, high extraversion, openness to experience, agreeableness and conscientiousness; exploration in breadth exhibits positive correlations with openness to experience, agreeableness, and conscientiousness; ruminative exploration is negatively correlated with emotional stability and extraversion; identification with commitment is linked to emotional stability, extraversion, conscientiousness, and openness to experience and finally commitment making tends to coincide with high extraversion, conscientiousness, openness to experience, and agreeableness. In the case of the identity formation styles, studies presented in the literature (Berzonsky and Sullivan 1992; Clancy Dollinger 1995; Dunkel et al. 2008; Duriez et al. 2004) suggest that the informational style is most closely related to high openness to experience, conscientiousness, and agreeableness; the normative style is positively correlated with conscientiousness and negatively with openness to experience; and finally the diffuse-avoidant style is associated with low consciousness, agreeableness, and openness to experience. Accordingly, one could assume that variables associated with Marcia’s (1966) exploration are connected to elements of the personality metatrait of plasticity: openness to experience and extraversion, where the relationships with openness to experience are prevailing. Further, variables rooted in Marcia’s (1966) commitment may be connected to the personality metatrait of stability: emotional stability, conscientiousness and agreeableness. In line with this, in the present research, we aim to investigate the relationships between basic identity formation categories (exploration and commitment) and basic personality metatraits (stability and plasticity).

## The Current Study

The main objective of the present study is to establish the trajectories of four identity formation modes (exploration vs. petrification and socialization vs. defiance) among early and middle adolescents, taking into account the personality underpinnings of modes development. In the literature, there is broad agreement that, in general, identity formation is the most dynamic during adolescence and emerging adulthood (Kroger 2017; Kroger et al. 2010; Marcia 1980; Meeus 2011). It is worth noting that identity formation during adolescence could be different during different periods of adolescence. Therefore, in order to more accurately examine these identity processes one can split this developmental period into early and middle or into early, middle and late adolescence. Early adolescence is the developmental stage when dealing with identity-relevant issues becomes particularly important (Kłym and Cieciuch 2015; Markovitch et al. 2017). Middle adolescence, in turn, is a period of typical teenage rebellion and in which the first independent decisions about future plans are typically made (Berzonsky 1982; Kroger 2017; Meeus et al. 2010). Although identity formation is viewed as having begun during early adolescence (Erikson 1959), the later stages are associated with the most dynamic identity development. One can expect that there will be qualitative differences in the course of identity formation changes and in the importance of particular identity formation variables. The majority of identity developmental research embraces middle-to-late adolescence and the college-age period as being where identity issues are of particular importance (see for example Kroger et al. 2010; Meeus 2011). Studies on the period of early adolescence as being where the identity formation process starts are in the minority (Hatano et al. 2016; Klimstra et al. 2010; Kłym and Cieciuch 2015; Markovitch et al. 2017; Meeus et al. 2012; Meeus et al. 2010). In our research, we conducted separate analyses to assess the trajectories of identity formation modes for early and middle adolescents. To describe the trajectories of identity formation modes more precisely, this study encompassed two cohorts: early and middle adolescents (approximately 13 and 16 years old, respectively) at the beginning of a new educational level (junior and senior high school, respectively).

To date, the longitudinal investigations of identity considers both the stability of status membership over time (Cramer 2017; Fadjukoff et al. 2016; Meeus et al. 2010) as well as changes in the mean-level of identity constructs. In the case of the five-processes model (Luyckx et al. 2006a), the findings are quite cohesive: exploration in depth, exploration in breath and commitment-making increase, but identification with commitment decreases linearly or changes quadratically in middle adolescence (Luyckx et al. 2006a, b, 2008). The findings concerning constructs from the model of three identity formation processes (Crocetti et al. 2008) are more varied. Pop et al. (2016) claim an increase in reconsideration of commitment in the whole sample, with differences in the intensity of changes between age groups and genders; in turn, commitment decreases similarly across the tested subgroups. Contradictory results are found by Crocetti et al. (2009), who show a decrease in reconsideration of commitment and an increase in commitment. Research in the area of vocational identity provides more ambiguity. In this case, identification with commitment, commitment making, exploration in breadth and exploration in depth decrease, and only variables associated with reconsideration of commitment increase (Negru-Subtirica et al. 2015). Klimstra et al. (2010) show that the courses of identity formation variables are different across age groups and genders. Although commitment achieves no significant change in any of the tested groups and exploration in depth increases among middle-to-late adolescents, the level of reconsideration of commitment among boys decreases among early-to-middle adolescents and increases among older participants (for a review, see Crocetti 2018).

Although the above-mentioned research concerns different models of identity formation than the present study which uses the circumplex of identity formation modes, the results build the basis for our expectations. The majority of research on adolescent identity development suggests an increase in exploration and a decrease in commitment (Crocetti et al. 2009; Luyckx et al. 2006a, b, 2008; Pop et al. 2016) but also some curvilinear trends among middle-to-late adolescents (Luyckx et al. 2008, 2014). Thus, we formulate the following hypotheses: in early adolescence, identity formation modes will change linearly such that petrification and socialization decrease and exploration and defiance increase (Hypothesis 1), and in middle adolescence, we assume the same pattern as in early adolescence, although some turning points are also possible which would result in curvilinear trends i.e., socialization can start to increase or exploration can start to decrease or maintain its level (Hypothesis 2).

As was discussed above, identity modes differentiated in the circumplex model capture, on the one hand, the meaning of exploration and commitment in the Marcia research tradition and, on the other hand, the meaning of the higher-order factors of personality (Cieciuch and Topolewska 2017). Based on the theoretical reasoning and empirical results obtained in research on personality-identity relations conducted within other models of identity discussed above (e.g. Dunkel et al. 2008; Luyckx et al. 2006a) we formulate the following expectations: stability is a positive predictor of socialization and a negative predictor of defiance, while plasticity is positive predictor of exploration and a negative predictor of petrification (Hypothesis 3). Taking into account that plasticity is responsible for personality dynamics, we expect that plasticity is a significant predictor of changes in identity formation modes (Hypothesis 4).

Additionally, gender is also considered an important factor for identity formation although the results are rather ambiguous. For instance, Luyckx et al. (2016) did not find a significant effect of gender on identity formation variables, while Crocetti et al. (2011) found that women explore more than men. Pop et al. (2016) found that girls have higher commitment and exploration that boys, however, according to Markovitch et al. (2017) boys had higher scores on commitment making. Taking into account previous research on gender and identity formation, we hypothesize that gender differentiates the level of the growth factors (Hypothesis 5). In summary, the contributions of the current project are (a) a description of the trajectories of identity modes growth in early and middle adolescence and (b) an explanation of both initial levels and change of identity modes by personality underpinnings in a way that is theoretically predicted by the circumplex of identity formation modes.

## Methods

### Participants and Procedure

The study was conducted in randomly selected Polish junior and senior high schools from the Mazovian region, which is rather homogeneous in terms of culture and ethnicity. Schools were contacted and invited to take part in our project. Five waves occurred between October 2015 and April 2017, with shorter breaks between the first, second and third measurement points (approximately 3 months) and longer breaks between the subsequent points (4 months). At the time of the first wave, all participants were attending the first year of junior or senior high school and completed on-line questionnaires at school in the presence of trained research assistants. The students received gifts for their participation. The present study has been approved by the Commission on Ethics and Bioethics at Cardinal Stefan Wyszyński University in Warsaw and has been performed in accordance with ethical standards. The study encompassed a total of 1,839 adolescents. The final sample consisted of *n* = 914 early adolescents (age at first wave *Mage* = 12.92, *SDage* = 0.47, 53.9% girls) and *n* = 925 middle adolescents (age at first wave *Mage* = 15.97, *SDage* = 0.34, 63.8% girls). Missing data for particular measurement points were as follows: first wave 17.3 and 16.2%; second wave 11.3 and 10.9%; third wave 17.7 and 27.9%; fourth wave 25.8 and 30.3%; and fifth wave 40.8 and 46.3% for early and middle adolescents, respectively. Little’s Missing Completely at Random (MCAR) test (1988) was used to check if data were missing completely at random. The results suggested that in both the early adolescent (*χ*^*2*^ = 284.30, df = 248, *p > *.05) and middle adolescent (*χ*^*2*^ = 261.78, df = 248, *p > *.05) groups as well as in the whole sample (*x*^*2*^ = 250.66, df = 248; *p > *.05), data were missing completely at random.

### Measures

#### Identity formation modes

The shortened version of the *Circumplex Identity Modes Questionnaire* (CIMQ; Topolewska and Cieciuch 2017) was used to measure the identity variables. This instrument consists of 16 items, 4 for each scale, corresponding to the four identity formation modes: socialization (I have clear and specific goals in life.), exploration (Getting involved in different things is a good way of getting to know myself.), defiance (Whenever I have to make a big decision about my life, I’m at a loss.), and petrification (I try to avoid situations which would force me to ask questions about myself.). The participants responded to the items on a Likert scale ranging from 1 (*not at all like me*) to 5 (*very much like me*). The Cronbach’s alpha values are reported in the Appendix (Tables 5–10).

#### Personality traits

The *Big Five Inventory* (BFI; John et al. 2008) was applied to assess the five personality traits during the second wave of the longitudinal research. This tool contains 46 items, ranging from 8 to 10 items per subscale, with the participants indicating their responses on a 5-point Likert scale ranging from 1 (*strongly disagree*) to 5 (*strongly agree*). The indicators of the metatraits stability and plasticity were regression-based factor scores obtained from exploratory factor analysis (EFA) on the five personality trait scales. The EFA parameters included principal axis factoring with two iterations and varimax rotation. This procedure was first applied by Digman (1997) and then replicated by other researchers (DeYoung et al. 2002; Strus and Cieciuch 2017). As a result, stability and plasticity representations were obtained. The factor loadings are presented in the Appendix (Tables 5–10).

### Analytical Strategy and Data Preparation

To verify our hypotheses, we conducted two series of latent growth curves (LGC). In the preliminary step, we tested the measurement invariance of identity formation modes over time. Because we were interested in analyzing means, we tested for the scalar measurement invariance (Cieciuch and Davidov 2014; Horn and McArdle 1992). In case full measurement invariance was not supported, we tested for partial scalar measurement invariance (Byrne et al. 1989). In order to assess whether the measurement invariance was established, we relied on the criteria proposed by Chen (2007). According to him, metric noninvariance is indicated by a change larger than .01 in CFI (comparative fit index), supplemented by a change larger than .015 in RMSEA (root mean square error of approximation) or a change larger than .03 in SRMR (standardized root mean square residual) compared with the configural invariance model. Regarding scalar invariance, noninvariance is indicated by a change larger than .01 in CFI, supplemented by a change larger than .015 in RMSEA or a change larger than .01 in SRMR compared with the metric invariance model.

The purpose of latent growth curve modeling is to assess the changes in the level of a construct over time. This method is based on two kinds of growth parameters: the *intercept*, which corresponds to the initial level of the construct, and *slopes*, which are indicators of the change rate across time. Latent growth curves make it possible to build models with a variety of change patterns, for example, linear, quadratic, cubic or piece-wise. Each growth factor is characterized by its mean and variance, where the mean refers to the significance of the particular parameter and variance is indicator of interindividual differences in parameter value. Latent growth curve models were run for each identity mode in both age groups.

To test the first and second hypotheses (pattern of change in identity formation modes among early and middle adolescents), a set of latent growth curve models with different growth patterns were tested to establish which solution best fits the data. We considered the following slopes: linear (systematic increase or decrease over time), quadratic (one curve collapse from the first to the last measurement point, i.e., an initial increase and subsequent decrease or vice versa) and cubic (the growth curve collapses over two time points, i.e., an initial growth, then a decrease, followed by growth again or vice versa). The model that best fit the data was chosen on the basis of the chi-square difference test. However, other model fit indices were also taken into account. Expected values are as follows: CFI ≥ .90, RMSEA < .05, and SRMR < .05 (Hu and Bentler 1999).

To test the third and fourth hypotheses (explaining identity formation by means of personality), personality metatraits were included in the final model as covariates predicting the initial level of the particular mode and the change factors. To test the fifth hypothesis, gender was also introduced as a covariate in the model. All analyses from the structural equation modeling approach were computed using *Mplus 7.2* software (Muthén and Muthén 1998–2012).

## Results

### Descriptive Statistics and Preliminary Analyses

Descriptive statistics, intercorrelations and Cronbach’s Alpha coefficients can be found in the Appendix (Tables 5–10). The results from the intra-class correlation analyses reveal that all modes are characterized by reliability over time, and the coefficients are as follows: exploration .787; socialization .858; petrification .844; and defiance .886. Because data were missing at random, we implemented the full information maximum likelihood (FIML) estimation.

Measurement invariance was analyzed using Mplus in multigroup confirmatory factor analysis where each wave in each age group was treated as a separate group. This method of analysis not only makes it possible to assess the measurement invariance between particular waves but also takes into account both of the tested cohorts. The results suggest that in the case of socialization, petrification and defiance, configural, metric and scalar levels of invariance were established in the analyzed data. For exploration, partial scalar measurement invariance was established (with the intercept of one item freely estimated). Thus, the precondition of latent growth curve modeling was confirmed, and we continued with the model estimation. The model fit coefficients of each measurement invariance level in multigroup confirmatory factor analysis are presented in the Appendix (Tables 5–10).

### Change in Identity Modes Over Time: Latent Growth Curve Modeling

**Fig. 1:**
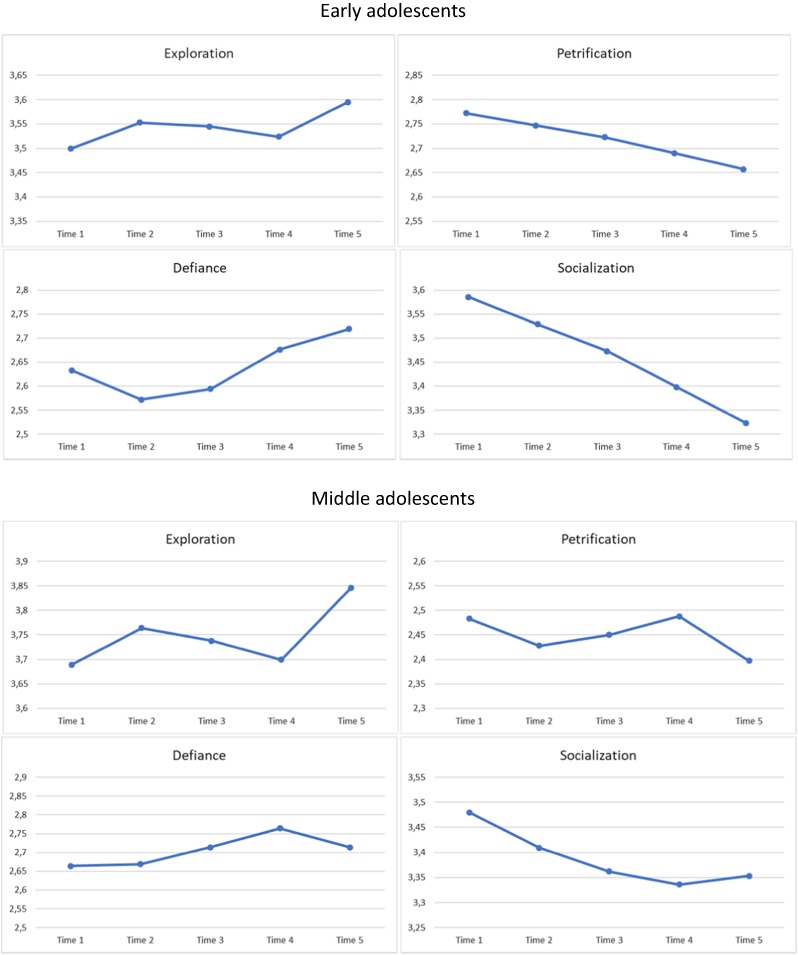
Trajectories of identity formation modes among early and middle adolescents

Table 2 presents the model fit coefficients of the latent growth curve models for each identity mode in both age groups and the result from the chi-square differences test conducted to compare models with different patterns of change. The results suggest that among early adolescents, the models with the best fit are those with the following change: socialization and petrification modes show a change with a linear pattern, while the trajectories of exploration and defiance best fit the models with nonlinear (cubic) trends. In turn, among middle adolescents, changes in all modes were nonlinear. More specifically, exploration, petrification and defiance trajectories had a cubic pattern, while socialization showed a quadratic pattern of change. Next, for each mode, in both age groups, we conducted separate latent growth curve analyses with the best fitting model. The results are presented in Table 3 and trajectories of identity formation modes with values of estimated means are presented in the Fig. 1.

**Table 2:** Model fit indices from LGC modeling among early and middle adolescents

		Chi^2^ (df)	CFI	RMSEA [90% CI]	SRMR	1. vs 2. Δ χ^2^ (Δ df)	1. vs 3. Δ χ^2^ (Δ df)	1. vs 4. Δ χ^2^ (Δ df)	2. vs 3. Δ χ^2^ (Δ df)	2. vs 4. Δ χ^2^ (Δ df)	3. vs 4. Δ χ^2^ (Δ df)
Early adolescents											
Exploration	Model 1.	68.56 (13)	0.893	.068 [.053; .085]	0.075	35 (3)**	36.27 (7) **	43.21 (8) **	1.27 (4)	8.21 (5)	6.94 (1)**
	Model 2.	33.56 (10)	0.955	.051 [.032; .070]	0.058						
	Model 3.	32.29 (6)	0.950	.069 [.047; .094]	0.056						
	Model 4. *m*	25.35 (5)	0.961	.067 [.042; .094]	0.064						
Socialization	Model 1.	95.49 (13)	0.892	.083 [.068; .099]	0.079	88.25**	90.77 (7) **	93.41 (12)**	2.52 (4)	5.16 (9)	2.64 (5)
	Model 2.	7.24 (10)	1.000	.000 [.000; .028]	0.025						
	Model 3.	4.72 (6)	1.000	.000 [.000; .037]	0.019						
	Model 4.	2.08 (1)	0.999	.034 [.000; .102]	0.009						
Petrification	Model 1.	55.17 (13)	0.937	.060 [.044; .076]	0.122	43.91 (3)**	50.43 (7)**	52.44 (8)**	6.52 (4)	8.53 (5)	2.01 (1)
	Model 2.	11.26 (10)	0.998	.012 [.000; .039]	0.047						
	Model 3.	4.74 (6)	1.000	.000 [.000; .038]	0.019						
	Model 4. *m*	2.73 (5)	1.000	.000 [.000; .033]	0.015						
Defiance	Model 1.	51.14 (13)	0.963	.057 [.041; .073]	0.057	19.72 (3)**	37.59 (7)**	51.12 (12)**	17.87 (4)**	31.40 (9)**	13.53 (5)**
	Model 2.	31.42 (10)	0.979	.048 [.030; .068]	0.051						
	Model 3.	13.55 (6)	0.993	.037 [.009; .064]	0.025						
	Model 4.	0.02 (1)	1.000	.000 [.000; .043]	0.001						
Middle adolescents											
Exploration	Model 1.	78.88 (13)	0.909	.074 [.059; .090]	0.131	17.61 (3)**	27.80 (7)**	60.68 (8)**	10.19 (4)*	43.07 (5)**	32.88 (1)**
	Model 2.	61.27 (10)	0.929	.074 [.057; .093]	0.116						
	Model 3.	51.08 (6)	0.937	.090 [.068; .114]	0.093						
	Model 4. *m*	18.20 (5)	0.982	.053 [.028; .081]	0.058						
Socialization	Model 1.	73.63 (13)	0.956	.071 [.056; .087]	0.083	50.45 (3)**	60.54 (7)**	62.58 (7)**	10.09 (4)*	12.13 (5)*	2.04 (1)
	Model 2.	23.18 (10)	0.990	.038 [.017; .058]	0.036						
	Model 3.	13.09 (6)	0.995	.036 [.007; .062]	0.015						
	Model 4. *m*	11.05 (5)	0.996	.036 [.000; .065]	0.012						
Petrification	Model 1.	45.13 (13)	0.969	.052 [.036; .069]	0.072	11.84 (3)**	13.4 (7)	27.61 (8)**	1.56 (4)	15.77 (5)**	14.21 (1)**
	Model 2.	33.29 (10)	0.977	.050 [.032; .070]	0.063						
	Model 3.	31.73 (6)	0.975	.068 [.046; .092]	0.061						
	Model 4. *m*	17.52 (5)	0.988	.052 [.027; .080]	0.045						
Defiance	Model 1.	55.95 (13)	0.969	.060 [0.44; .076]	0.044	33.90 (3)**	41.12 (7)**	47.15 (8)**	7.22 (4)	13.25 (5)**	6.03 (1)*
	Model 2.	22.05 (10)	0.991	.036 [.015; .057]	0.026						
	Model 3.	14.83 (6)	0.994	.040 [.014; .066]	0.025						
	Model 4. *m*	8.80 (5)	0.997	.029 [.000; .059]	0.024						

*CFI* comparative fit index, *RMSEA* root mean square error of approximation, *CI* confidence interval, *SRMR* standardized root mean square residual, *Model 1.* model with intercept only, *Model 2.* model with intercept and linear growth factor, *Model 3.* model with intercept, linear and quadratic growth factors, *Model 4.* model with intercept, linear, quadratic and cubic growth factors, *m*because of a non-positive covariance matrix at least one growth factor’s variance was fixed to 0

*p < .05; **p < .01

**Table 3:** Growth coefficients of Latent Growth Curve models among early and middle adolescents

	Intercept		Linear		Quadratic		Cubic	
	*M*	*V*	*M*	*V*	*M*	*V*	*M*	*V*
Early adolescents								
Exploration	3.499***	.180***	.133*	0.05	−.097*	.002	.019**	.000*m*
Socialization	3.586***	.284***	−.075***	.015***	–	–	–	–
Petrification	2.772***	.172***	−.033***	.011**	–	–	–	–
Defiance	2.633***	.414***	−.157*	.962*	.114*	.465*	−.018	0.015
Middle adolescents								
Exploration	3.689***	.133***	.205	0.052	−.167***	.004	.035***	.000*m*
Socialization	3.480***	.345***	−.110***	0.001	.021**	.001	–	–
Petrification	2.483***	.286***	−.153**	.000*m*	.125**	.014	−.025**	.001
Defiance	2.664***	.434***	−.040	.000*m*	.073	.026*	−.016*	.002*

*M* mean, *V* variance, *m* because of a non-positive covariance matrix at least one growth factor’s variance was fixed to 0

*p < .05; **p < .01; ***p < .001

In the younger group, exploration generally increases from T1 to T5, but the pattern of change is also characterized by quadratic and cubic trends. This finding suggests that initially the level of exploration increases; then, from T2 to T3, it decreases; and, subsequently, from T3 to T5, it increases again. Both, the petrification and socialization modes decrease linearly from T1 to T5. Although at the stage of model comparison, in the case of defiance, the model with the cubic trend proved best fit the data, the mean of the cubic slope was not significant. Finally, the defiance mode could be characterized as generally increasing from T1 to T5 but with a trajectory taking a quadratic U-shaped pattern. Among middle adolescents, the identity formation mode trajectories are slightly different. In general, the exploration level is similar in T1 and T5 (non-significant linear slope), but it changes according to the nonlinear trend, with an initial increase, followed by a decrease from T2 to T3 and then again increases. Although the socialization mode decreases from T1 to T5, in contrast to the early adolescents, the trajectory takes nonlinear quadratic pattern, with an initial decrease followed by an increase from T3 to T5. For the petrification mode change characterized by polyline (an initial decrease, then an increase from T2 to T4 and, finally, a decrease). In the last of the tested modes, defiance changes non-linearly, with an increase to T3, a somewhat stable level to T4 and then a decrease again. The result met our expectations. In early adolescence, socialization and petrification decrease, while exploration and defiance generally increase, although quadratic and cubic changes are also found. Among middle adolescents, all modes change with a non-linear pattern: Socialization starts to increase from T3, exploration and defiance levels fluctuate but in general maintain their levels. Moreover, the intercepts of all tested variables within the two age groups and some of the slopes show significant variance, which indicates interindividual differentiation in the participants’ performance. In line with our assumptions, we introduce personality metatraits to explain this internal variation in both of the tested groups.

### The Role of Personality Higher-Order Factors and Gender: Conditional Latent Growth Curve Modeling

The conditional latent growth curve models with stability and plasticity metatraits and gender as predictors of the intercept and slopes are estimated for each of the identity formation modes in both age groups. The results are presented in Table 4. In line with the hypothesis that personality metatraits would predict the initial levels of identity formation modes, in both early and middle adolescence, stability predicted socialization positively and defiance negatively; plasticity predicted exploration positively and petrification negatively. The hypothesis that plasticity would be a predictor of changes in identity formation modes was also confirmed. The results suggest that plasticity predicts a positive linear change in exploration and a negative linear change in socialization and petrification but only in early adolescents. Moreover, the effect of plasticity on quadratic (negative) and cubic (positive) growth parameters is significant. This finding means that plasticity is not only associated with linear growth in exploration from the first to the last measurement occasion but also with different changes in exploration in early adolescence, with an initial increase (linear), then a decrease (quadratic), followed again by an increase (cubic).

**Table 4:** Standardized path coefficients from conditional LGC

		Stability	Plasticity	Gender
Early adolescents				
Exploration	Intercept	0.128	0.520***	0.001
	Linear	0.019	0.820*	−0.358
	Quadratic	0.186	−0.152***	0.557
	Cubic	−0.309	1.161***	−0.734
Socialization	Intercept	0.520***	0.510***	0.390***
	Linear	−0.020	−0.360**	−0.240
Petrification	Intercept	−0.420***	−0.186*	0.270*
	Linear	0.198	−0.426**	−0.078
Defiance	Intercept	−0.337***	−0.116**	−0.051
	Linear	−0.109	−0.136	−0.307*
	Quadratic	0.068	0.109	0.199
	Cubic	−0.008	−0.021	−0.032
Middle adolescents				
Exploration	Intercept	0.254**	0.770***	−0.066
	Linear	−0.174	0.179	−1.069
	Quadratic	−0.106	−0.328	1.494*
	Cubic	0.514	0.293	−1.939**
Socialization	Intercept	0.800***	0.250***	0.230**
	Linear	0.000	−0.030	−0.300
	Quadratic	−0.130	−0.140	0.310
Petrification	Intercept	−0.123**	−0.196***	0.117*
	Linear	−0.026	−0.044	−0.041
	Quadratic	0.047	0.047	−0.045
	Cubic	−0.011	−0.009	0.018
Defiance	Intercept	−0.568***	−0.133**	−0.114*
	Linear	0.013	−0.049	−0.058
	Quadratic	0.031	0.050	0.009
	Cubic	−0.008	−0.009	0.004

*Note*: Standardized coefficients under the STDY standardization type. Females are coded as 0 and males as 1

*p < .01; **p < .005; ***p < .001

To verify the fifth hypothesis (the role of gender), we examined whether gender predicted the initial levels and growth indicators of identity formation modes. Among early adolescents, males show a higher initial level of socialization and petrification and a lower linear change rate of defiance compared to females. In the group of middle adolescents, males were also higher in their initial level of socialization and petrification but also had a lower level of defiance. Moreover, being male predicts a non-linear change in exploration (positive in the case of quadratic and negative in the case of cubic). In summary, we can say that in addition to the personality stability and plasticity metatraits, gender is a significant predictor of the initial level of identity formation modes in particular.

## Discussion

The present study aimed to examine the trajectories of identity formation modes among early and middle adolescents with regard to the personality underpinnings of variability in identity formation. Although the interplay of personality and identity has been addressed in some previous longitudinal research (Hatano et al. 2016; Luyckx et al. 2006a, b; Luyckx et al. 2014), to the best of our knowledge, no study to date has investigated the trajectories of identity formation variables with personality traits as predictors of change in identity variables among early and middle adolescents separately. The present study is the first longitudinal investigation of the identity formation modes differentiated in the circumplex model that was developed as an integrative model of identity formation encompassing several existing models (Cieciuch and Topolewska 2017).

In line with previous research, identity formation modes have been found to change over time. In this present study, all four identity formation modes changed significantly during adolescence, among both early and middle adolescents. Although the direction of changes was generally similar in the two age groups (decrease in socialization and petrification and increase in exploration), with a more fine-grained analysis, one can observe differences in patterns of changes. Among early adolescents, the trajectory of socialization decreases linearly, while in the older group, change is nonlinear. More specifically, among middle adolescents change in socialization shows a quadratic trend that starts to increase at the third measurement point (6 month after initial measurement). Additionally, among early adolescents, exploration generally increases over time with two bends in the middle. Whereas among middle adolescents, exploration is characterized by nonlinear change with two turning points at the second and fourth measurement occasion. Therefore, it seems that in exploration grows at the beginning of adolescence, while in middle adolescence it fluctuates by alternately increasing and decreasing. The largest difference between the two age groups was found in defiance. In early adolescents, defiance increases linearly after an initial decrease. In middle adolescents the trend is far more curvilinear (cubic), showing stability for the first (3 months after initial measurement), followed by an increase and, finally, a decrease (from fourth to fifth measurement occasion). Petrification decreases linearly in the younger group but is nonlinear with two turning points among middle adolescents. Indeed, our research design with five measurement points over 1.5 years with three-four months intervals allowed us to provide these detailed analyses of growth patterns.

The results could shed new light on inconsistent findings from previous research on identity formation variables growth during adolescence (Crocetti et al. 2009; Hatano et al. 2016; Klimstra et al. 2010; Luyckx et al. 2008; Negru-Subtirica et al. 2015; Pop et al. 2016). Firstly, trajectories of identity formation constructs depend on the adolescents’ age. In our results, the tendency to explore generally increases among younger adolescents, while among middle adolescents the level of exploration changes many times during the two-year period. The socialization mode unambiguously decreases among early adolescents, but around seventeens among middle adolescents it starts to increase with time. Most research on identity development concerns middle and late adolescence and emerging adulthood (see Kroger et al. 2010; Meeus 2011). Moreover, these studies do not always provide comparisons of the results for the different age groups. Nevertheless, our comparison age groups shows that early and middle adolescents differ in their engagement with identity formation. While early adolescence is viewed as the moment when the identity formation process becomes extraordinarily important, during middle and late adolescence this process is more advanced and complicated. The strong decline of socialization among early adolescents and the U-shaped trajectory of this mode among middle adolescents indicated differences in the identity structure between two age groups. Among younger adolescents, doubts about personal identity seem to be growing and dynamic changes in identity modes are increasing. In the group of middle adolescents, uncertainty seems to stabilize and transform around the age of seventeen into the first conscious identity commitments. Given the number of dynamic changes found in our results, as well as the number of differences between early and middle adolescents, it could be suggested that analyses that cover the entire period of adolescence without taking into account potential developmental differences between early and middle adolescents could lead to unclear results.

By embracing multiple measurements points with short intervals our design allows us to show curvilinear changes which could be unnoticed with less interval intensive data or with longer breaks between measurement occasions. For example, taking into account only first, third and fifth measurements of exploration in both early and middle adolescence would only show linear growth. Similarly, using the same three measurement points, one may conclude that there was no significant change in defiance at all, especially in the group of middle adolescents. In sum, separate analyses for early and middle adolescents and a research approach with five measurement points with short intervals revealed that the tendencies to explore the domain of identity (exploration) increased among the youngest and fluctuated among the older adolescents. Further, it also revealed that the tendency to fulfill social roles in a coherent and stable way (high socialization and low defiance) strongly decreased among younger adolescents but eventually increased around the seventeen of older adolescents.

With regards to the broader personality structure, identity formation modes are located in the layer of characteristic adaptations and are influenced by personality traits and metatraits belonging to the dispositional trait layer, which is more resistant to change (McAdams 1995; McAdams and Pals 2006). The implication is that personality provides a stable basis for the creation of more dynamic and changeable constructs, such as identity. While throughout the human lifespan, personality traits generally remain quite stable and change only slightly, changes in identity variables occur more readily and dynamically. The results from our study show that these changes are in line with the classical approach of Marcia (1966) and that the role of personality metatraits is consistent with the theoretical assumptions about personality underpinnings made by Cieciuch and Topolewska (2017). In other words, the stability personality metatrait, as a basic force for maintaining cohesion, serves as a basis for the socialization vs. defiance dimension (analogous to identity commitment dimension in the Marcia’s 1966 model), while the plasticity personality metatrait is responsible for dynamic adaptation to new circumstances and personal growth, thus underpinning exploration vs. petrification dimension (analogous to identity exploration in the Marcia 1966 model). Moreover, the strength of the predictions, as well as simple correlations between personality metatraits and identity formation modes reveal additional differences between early and middle adolescents. For instance, socialization in middle adolescents is explained predominantly by stability, however, in early adolescents both plasticity and stability are related to the initial level. Plasticity as a factor of personal growth positively predicts the level of identity stability in the beginning of the identity formation process, before the typical period of identity questioning begins.

The role of personality metatraits is also significant in the course of changes in identity formation modes. The plasticity metatrait predicts the rate of change in identity formation modes, but only in early adolescence. Thus, personality disposition and plasticity in particular enhance the change in identity formation during the period when identity formation is about to start. When identity formation is advanced and the most dynamic (middle adolescence), personality is no longer responsible for the intensity of the change rate. In other words, the plasticity personality metatrait catalyzes identity development during early adolescence, but the role of this metatrait declines during middle adolescence when identity becomes somewhat independent from the dispositional personality located in the first layer of personality structure (McAdams and Manczak 2011; McAdams and Pals 2006).

Our research shows gender differences in identity formation modes. Boys have a higher tendency for identity stability (socialization) and are more willing to maintain their current identity structure (petrification) compared to girls. Markovitch et al. (2017) found similar results; however, Pop et al. (2016) found that girls were higher in identity commitment. In the case of slopes, among early adolescents linear (increasing) change was stronger among girls, and among middle adolescents, girls also had more change. Taking into account the general decreasing tendency of socialization and different timing with regards to developmental changes such as puberty, these results could imply that boys had higher levels of socialization compared to girls, because their identity development is less advanced that girls at the same age.

The present study encompassed a shorter than two-year period, during which five measurements were carried out. The three- or four-month intervals between consecutive waves enabled a precise evaluation of changes; nevertheless, the overall study period is somewhat short. Although identity formation is considered to be most dynamic during adolescence, it should be investigated in the short term as well as over longer periods of time (several years), considering the lifelong relevance of identity development.

Additionally, the longitudinal growth analyses provided the variance level of the intercepts and slopes which informs us about individual differences in growth factors of the tested constructs. The significance values of the intercept variance suggest that both early and middle adolescents differ in the initial levels of their identity formation modes. Socialization (only in the group of early adolescents), petrification and defiance also show a significant variance in at least one slope, which indicates that identity formation modes have variant trajectories of change during adolescence. These interindividual differences in the course of the change in identity formation modes require a more detailed investigation aimed at finding identity formation trajectories incorporating all modes simultaneously.

## Conclusions

The present research uses the circumplex of identity formation modes as an integrative model of identity formation to describe identity development in early and middle adolescents. All identity formation modes changed, however differently for each age group. Early adolescents were characterized by a decrease in petrification and socialization and a general increase in exploration and defiance. Middle adolescents showed primarily nonlinear patterns of change with some bends in the middle, indicating more dynamics and shifts in the identity development process compared to younger participants. The stability and plasticity personality metatraits were significant predictors of the initial level of identity formation modes. More specifically, stability predicted identity socialization, while plasticity underpinned identity exploration. Additionally, the plasticity metatrait positively predicts the change in identity formation modes among early adolescents. Boys show a higher level of socialization and petrification as well as a lower level of defiance compared to girls. Thus, the circumplex of identity formation allows for the description of both the trajectories of identity development and the personality underpinnings of this process.

## Appendix

**Table 5:** Means and standard deviations of identity formation modes

	Time 1		Time 2		Time 3		Time 4		Time 5	
	M	SD	M	SD	M	SD	M	SD	M	SD
*Early adolescents*										
Exploration	3.48	0.61	3.59	0.59	3.51	0.60	3.57	0.61	3.61	0.64
Defiance	2.60	0.80	2.56	0.82	2.59	0.83	2.66	0.85	2.69	0.82
Petrification	2.77	0.71	2.72	0.69	2.71	0.71	2.68	0.71	2.65	0.70
Socialization	3.57	0.75	3.54	0.75	3.47	0.75	3.42	0.70	3.31	0.74
*Middle adolescents*										
Exploration	3.70	0.57	3.82	0.51	3.72	0.58	3.75	0.55	3.86	0.55
Defiance	2.66	0.84	2.67	0.83	2.75	0.82	2.76	0.84	2.72	0.85
Petrification	2.49	0.70	2.37	0.72	2.44	0.69	2.46	0.71	2.38	0.76
Socialization	3.48	0.79	3.45	0.76	3.30	0.78	3.35	0.81	3.37	0.81

**Table 6:** The coefficients from r-Pearson correlation analysis among early adolescents

		1	2	3	4	5	6	7	8	9	10	11	12	13	14	15	16	17	18	19	20	21
1	Exploration T1	–																				
2	Exploration T2	.34**	–																			
3	Exploration T3	.32**	.42**	–																		
4	Exploration T4	.27**	.28**	.37**	–																	
5	Exploration T5	.29**	.38**	.45**	.47**	–																
6	Socialization T1	.41**	.23**	.19**	.17**	.10*	–															
7	Socialization T2	.21**	.35**	.21**	.20**	.14**	.48**	–														
8	Socialization T3	.13**	.18**	.36**	.15**	.16**	.41**	.48**	–													
9	Socialization T4	.15**	.14**	.11*	.35**	.15**	.43**	.42**	.50**	–												
10	Socialization T5	.07	.16**	.19**	.20**	.34**	.38**	.39**	.43**	.51**	–											
11	Petrification T1	.05	−.04	−.18**	−.21**	−.19**	.02	.03	−.02	−.05	−.14**	–										
12	Petrification T2	−.18**	−.14**	−.16**	−.25**	−.26**	−.17**	−.16**	−.13**	−.08	−.17**	.34**	–									
13	Petrification T3	−.08	−.17**	−.18**	−.26**	−.24**	−.09*	−.13**	−.18**	−.20**	−.20**	.32**	.42**	–								
14	Petrification T4	−.10*	−.19**	−.26**	−.22**	−.28**	−.11*	−.11**	−.19**	−.13**	−.12**	.36**	.44**	.51**	–							
15	Petrification T5	−.12*	−.20**	−.23**	−.32**	−.28**	−.17**	−.13**	−.14**	−.15**	−.15**	.31**	.45**	.49**	.56**	–						
16	Defiance T1	−.01	−.08	−.12**	−.16**	−.12*	−.40**	−.28**	−.28**	−.32**	−.34**	.46**	.27**	.30**	.35**	.24**	–					
17	Defiance T2	−.12**	−.10**	−.12**	−.16**	−.11*	−.36**	−.47**	−.37**	−.32**	−.32**	.13**	.47**	.32**	.26**	.26**	.51**	–				
18	Defiance T3	−.09*	−.07	−.15**	−.19**	−.09	−.30**	−.35**	−.52**	−.41**	−.37**	.18**	.24**	.48**	.30**	.22**	.47**	.58**	–			
19	Defiance T4	−.06	−.09*	−.18**	−.10**	−.12**	−.34**	−.28**	−.39**	−.44**	−.37**	.19**	.19**	.35**	.44**	.28**	.52**	.48**	.57**	–		
20	Defiance T5	−.08	−.15**	−.19**	−.25**	−.20**	−.35**	−.31**	−.33**	−.44**	−.54**	.24**	.31**	.35**	.30**	.45**	.51**	.50**	.53**	.58**	–	
21	Stability	.15**	.19**	.23**	.21**	.15**	.33**	.41**	.37**	.37**	.30**	−.17**	−.23**	−.23**	−.21**	−.15**	−.43**	−.47**	−.44**	−.36**	−.33**	–
22	Plasticity	.27**	.42**	.34**	.31**	.32**	.30**	.33**	.22**	.19**	.22**	−.10*	−.26**	−.20**	−.27**	−.30**	−.23**	−.25**	−.21**	−.18**	−.21**	.30**

*Note*: T1-T5 = first, second, third, fourth and fifth measurement occasions, respectively

**p* < .05; ***p* < .01

**Table 7:** The coefficients from r-Pearson correlation analysis among middle adolescents

		1	2	3	4	5	6	7	8	9	10	11	12	13	14	15	16	17	18	19	20	21
1	Exploration T1	–																				
2	Exploration T2	.42**	–																			
3	Exploration T3	.41**	.48**	–																		
4	Exploration T4	.40**	.46**	.52**	–																	
5	Exploration T5	.40**	.42**	.44**	.51**	–																
6	Socialization T1	.35**	.20**	.12**	.19**	.17**	–															
7	Socialization T2	.21**	.27**	.11**	.18**	.17**	.61**	–														
8	Socialization T3	.23**	.24**	.31**	.24**	.18**	.56**	.62**	–													
9	Socialization T4	.22**	.20**	.21**	.29**	.16**	.58**	.65**	.64**	–												
10	Socialization T5	.20**	.23**	.23**	.17**	.26**	.52**	.60**	.66**	.68**	–											
11	Petrification T1	−.30**	−.27**	−.36**	−.29**	−.27**	−.13**	−.14**	−.23**	−.15**	−.19**	–										
12	Petrification T2	−.34**	−.34**	−.34**	−.28**	−.31**	−.18**	−.15**	−.18**	−.22**	−.18**	.53**	–									
13	Petrification T3	−.26**	−.31**	−.38**	−.35**	−.32**	−.11*	−.12**	−.23**	−.22**	−.27**	.55**	.60**	–								
14	Petrification T4	−.27**	−.29**	−.33**	−.37**	−.32**	−.19**	−.16**	−.27**	−.25**	−.25**	.42**	.49**	.53**	–							
15	Petrification T5	−.24**	−.32**	−.39**	−.37**	−.40**	−.15**	−.18**	−.22**	−.22**	−.21**	.47**	.57**	.53**	.57**	–						
16	Defiance T1	−.13**	−.17**	−.13**	−.14**	−0.1	−.60**	−.55**	−.50**	−.50**	−.46**	.37**	.23**	.25**	.20**	.15**	–					
17	Defiance T2	−.19**	−.17**	−.10*	−.12**	−.15**	−.55**	−.64**	−.54**	−.53**	−.50**	.23**	.37**	.26**	.22**	.24**	.62**	–				
18	Defiance T3	−.10*	−.14**	−.15**	−.11*	−.14**	−.47**	−.54**	−.62**	−.58**	−.60**	.23**	.23**	.35**	.21**	.20**	.62**	.68**	–			
19	Defiance T4	−.16**	−.15**	−.15**	−.17**	−.14**	−.44**	−.52**	−.56**	−.69**	−.60**	.24**	.24**	.28**	.42**	.30**	.53**	.58**	.66**	–		
20	Defiance T5	−.15**	−.21**	−.23**	−.16**	−.17**	−.43**	−.56**	−.53**	−.60**	−.69**	.22**	.24**	.29**	.25**	.39**	.49**	.60**	.61**	.65**	–	
21	Stability	.20**	.17**	.10*	.12**	.14**	.49**	.52**	.42**	.47**	.41**	−.17**	−.16**	−.15**	−.09*	−0.07	−.52**	−.54**	−.48**	−.46**	−.43**	–
22	Plasticity	.32**	.39**	.25**	.32**	.26**	.24**	.27**	.19**	.22**	.15**	−.22**	−.24**	−.20**	−.17**	−.17**	−.24**	−.27**	−.21**	−.21**	−.19**	.30**

*Note*: T1-T5 = first, second, third, fourth and fifth measurement occasions, respectively

**p* < .05; ***p* < .01

**Table 8:** Cronbach’s alpha coefficients

	Exploration		Socialization		Petrification		Defiance	
	EA	MA	EA	MA	EA	MA	EA	MA
Time 1	.52	.58	.66	.74	.55	.58	.71	.73
Time 2	.53	.47	.68	.71	.52	.60	.71	.71
Time 3	.55	.53	.69	.73	.55	.59	.71	.73
Time 4	.59	.55	.64	.77	.60	.66	.76	.77
Time 5	.61	.56	.69	.77	.55	.69	.74	.75

*EA* early adolescents, *MA* middle adolescents

**Table 9:** The factor loadings from the exploratory factor analysis on the scales of five personality traits

	Early adolescents		Middle adolescents	
	Factor 1. (stability)	Factor 2. (plasticity)	Factor 1. (stability)	Factor 2. (plasticity)
Neuroticism	−.662	−.036	−.587	−.048
Conscientiousness	.641	.447	.474	.195
Agreeableness	.490	.347	.497	.157
Openness to Experience	.079	.658	.051	.529
Extraversion	.208	.469	.323	.495

**Table 10:** The fit indicators from the measurement invariance test

	Chi2 (df)	CFI	RMSEA	SRMR
Exploration				
Configural	75.14 (20)	.971	.064 (.049; .080)	.022
Metric	123.69 (47)	.960	.049 (.039; .060)	.040
Scalar^a^	163.66 (65)	.948	.048 (.039; .057)	.044
Socialization				
Configural	416.174 (20)	.923	.172 (.158; .187)	.045
Metric	463.707 (47)	.919	.115 (.106; .125)	.056
Scalar	526.247 (74)	.912	.096 (.088; .104)	.058
Petrification				
Configural	103.076 (20)	.965	.079 (.064; .094)	.024
Metric	140.339 (47)	.961	.055 (.044; .065)	.034
Scalar	193.940 (74)	.950	.049 (.041; .058)	.039
Defiance				
Configural	112.684 (20)	.983	.083 (.069; .099)	.021
Metric	157.466 (47)	.979	.059 (.049; .070)	.037
Scalar	235.839 (74)	.970	.057 (.049; .066)	.041

^a^ Scalar measurement was partial—one parameter is freely estimated across groups
